# Effects of Stellate Ganglion Block Through Different Approaches Under Guidance of Ultrasound

**DOI:** 10.3389/fsurg.2021.797793

**Published:** 2022-01-17

**Authors:** Hai-Hua Shan, Hong-Fang Chen, Yong Ni, Jia-Xuan Yang, Xue-Lan Zhou

**Affiliations:** Department of Anesthesiology, The Second Affiliated Hospital of Soochow University, Suzhou, China

**Keywords:** ultrasonography, approach, stellate ganglion, nerve block, safety, effectiveness

## Abstract

**Objective:**

This study aimed to investigate the effects of stellate ganglion block (SGB) through different approaches under guidance of ultrasound.

**Methods:**

A total of 130 patients undergoing SGB in our hospital between February 2019 and February 2020 were enrolled as the research subjects. According to the random number table method, these subjects were divided into two groups: a modified 6th cervical vertebra (C6) group (*n* = 65) and a 7th cervical vertebra (C7) group (*n* = 65). Under the guidance of ultrasound, the subjects in the modified C6 group were punctured at the level of the C6 transverse process, and the subjects in the C7 group were punctured at the level of the C7 transverse process. The operation duration, number of puncture angle adjustments, block effects, and adverse reactions for SGB were compared between the two groups.

**Results:**

The modified C6 group showed shorter SGB operation duration and a lower number of puncture angle adjustments than the C7 group, and the differences were statistically significant (*P* < 0.05). Horner Syndrome occurred in both groups after SGB. The incidence of adverse reactions in the modified C6 group was 4.62%, comprising 1 case of hoarseness and 2 cases of slowed pulse, while that in the C7 group was 6.15%, with 1 case of hoarseness and 3 cases of slowed pulse; the difference between the two groups was not statistically significant (*P* > 0.05).

**Conclusion:**

The operation duration for modified SGB guided by ultrasound puncturing at the C6 transverse process is shorter and requires fewer puncture angle adjustments than puncturing at the C7 transverse process; however, there is no significant difference between the incidence of adverse reactions or the blocking effects of the two methods.

## Introduction

Stellate ganglion block (SGB) can regulate the autonomic nervous and cerebrovascular systems, dilate blood vessels, and improve circulation; therefore, it is widely used in the treatment of head, neck, and upper limb pain induced by multiple factors (1). Ding et al. (2) reported that ultrasound-guided SGB is more accurate than blind exploration. However, there are differing reports on ultrasound-guided SGB through lateral approaches, including blocking at the 7th cervical vertebra (C7) transverse process and at the 6th cervical vertebra (C6) transverse process (3, 4). There are few comparative studies regarding puncturing at the C6 and C7 levels. As the anterior tubercle of the C6 transverse process is long (5), the gap between the anterior tubercle and the internal jugular vein is very narrow (<5 mm), which affects the needle insertion. Therefore, this study innovatively adopted the modified C6 transverse process inferior approach: when the ultrasound screen clearly exhibited the C6 transverse process and its anterior and posterior tubercles, the ultrasound probe was slightly moved toward C7, and when the anterior tubercle of the C6 transverse process disappeared from the screen, the needle was inserted to complete the operation. This paper reports the observed effects of SGB using these two puncture approaches.

## Materials and Methods

### General Information

This study was conducted in accordance with the declaration of Helsinki and approved by the ethics committee of the Second Affiliated Hospital of Soochow University (ethics No.: JD-LK-2018-103-01), and all patients signed informed consent to participate in the study. A total of 130 patients who underwent SGB treatment in our hospital between February 2019 and February 2020 were enrolled as the research subjects, and the participants were divided into two groups according to the random number table method: a modified C6 group (*n* = 65) and a C7 group (*n* = 65). In the modified C6 group, there were 33 males and 32 females, the average age was 38.5 ± 10.2 years, and the average body weight was 62.8 ± 8.0 kg. This group contained 23 patients with cervical spondylosis, 40 patients with headache, and 2 patients with dysmenorrhea. In the C7 group, there were 35 males and 30 females, the average age was 38.2 ± 10.6 years, and the average body weight was 62.3 ± 8.2 kg. This group contained 25 patients with cervical spondylosis, 37 patients with headache, and 3 patients with dysmenorrhea.

### Inclusion and Exclusion Criteria

Inclusion criteria (6): (1) no abnormality in neck movement; (2) no trauma or infection at the puncture site; (3) normal coagulation function; (4) ASA grade I–II. Exclusion criteria (7): (1) history of neck surgery; (2) bleeding disease or other puncture contraindications; (3) complications involving serious disease of the heart, lung, kidneys, or other organs; (4) occurrence of glaucoma, atrioventricular block, or acute myocardial infarction within the previous month.

### Methods

**Figure 1:**
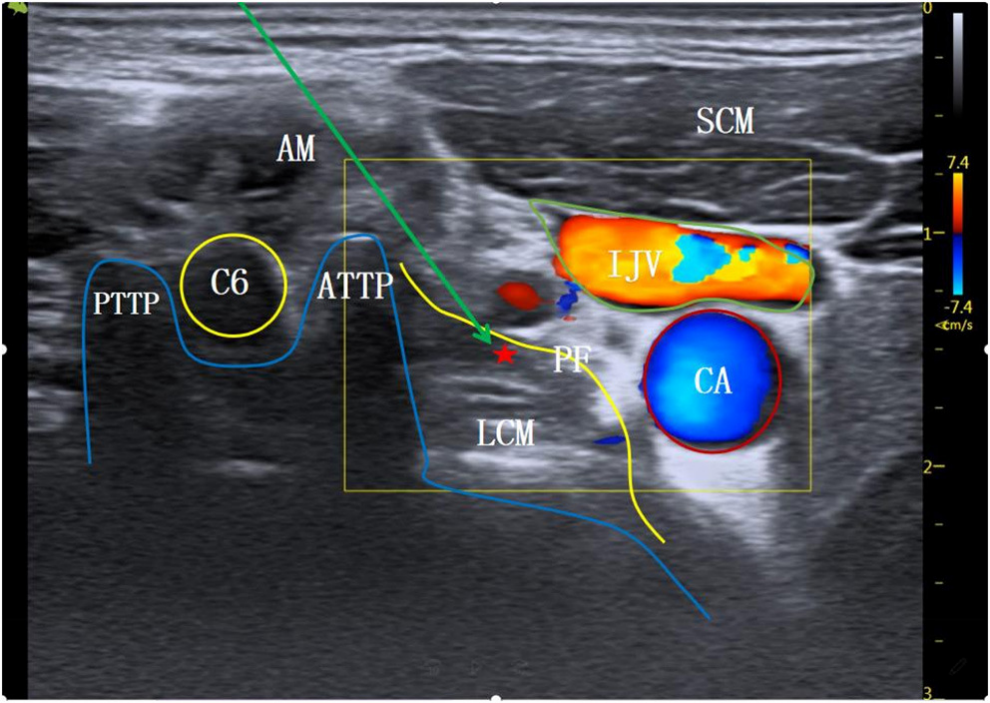
The level at C6 transverse process. AM, anterior scalene muscle; SCM, sternocleidomastoid muscle; C6, the 6th cervical nerve root; ATTP, anterior tubercle of transverse process; PTTP, posterior tubercle of transverse process; PF, anterior vertebral fascia; LCM, long neck muscle; ⋆Indicates stellate ganglion block area; ↘Indicates simulated puncture path; IJV, internal jugular vein; CA, carotid artery.

**Figure 2:**
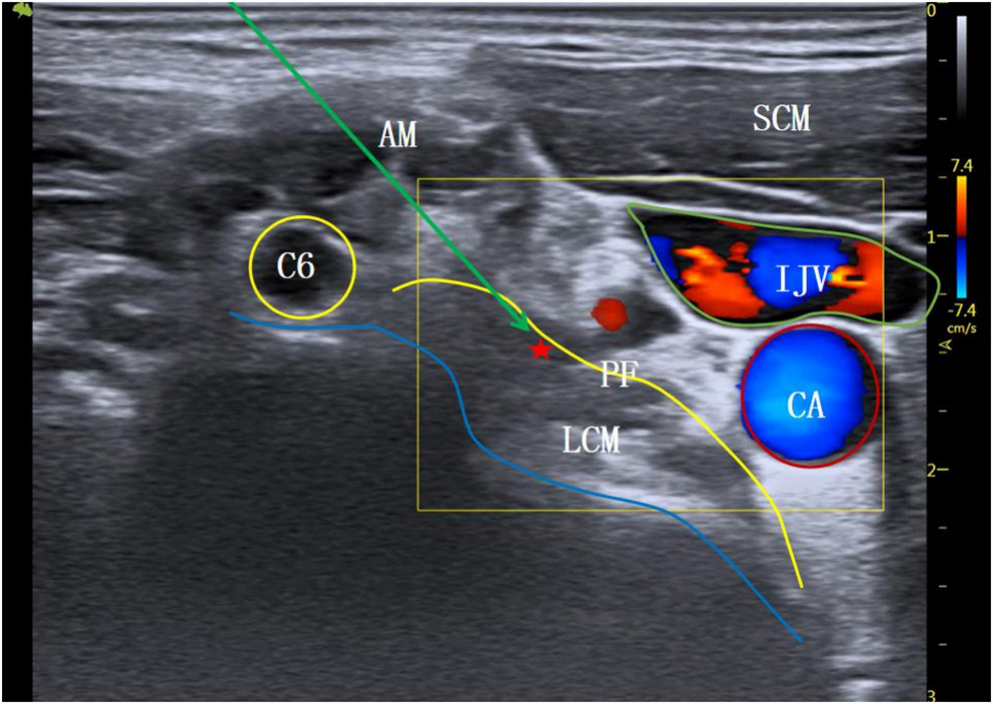
Modified inferior C6 transverse process level. AM, anterior scalene muscle; SCM, sternocleidomastoid muscle; C6, the 6th cervical nerve root; PF, anterior vertebral fascia; LCM, long neck muscle; ⋆Indicates stellate ganglion block area; ↘Indicates simulated puncture path; IJV, internal jugular vein; CA, carotid artery.

**Figure 3:**
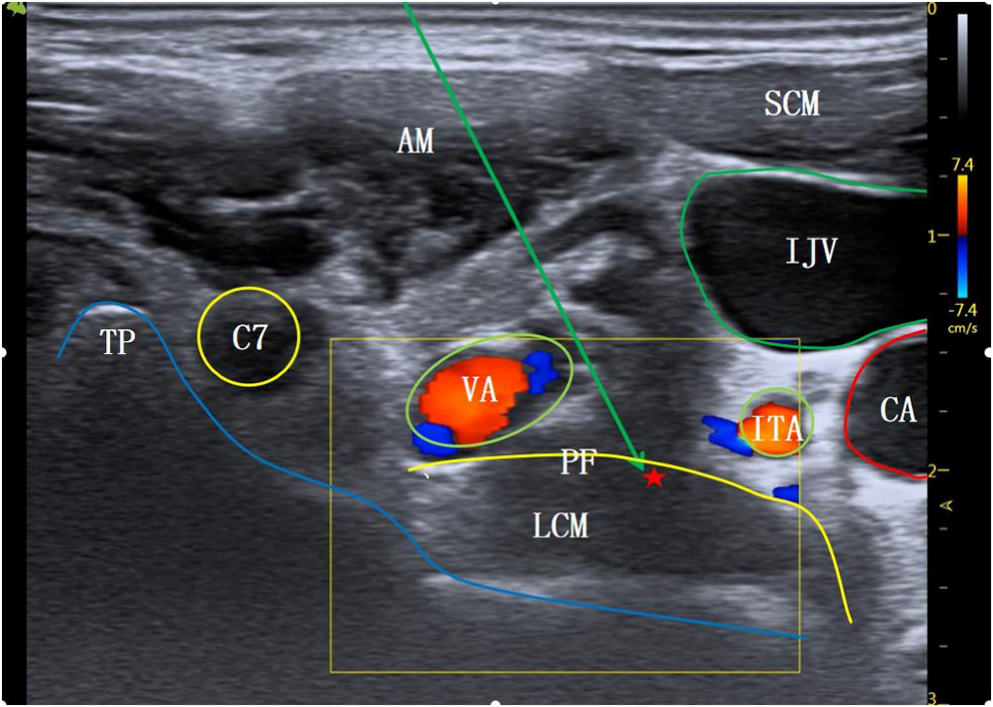
The level at C7 transverse process. AM, anterior scalene muscle; SCM, sternocleidomastoid muscle; C7, the 7th cervical nerve root; TP, transverse process; VA, vertebral artery; ITA, inferior thyroid artery; PF, anterior vertebral fascia; LCM, long neck muscle; ⋆Indicates stellate ganglion block area; ↘Indicates simulated puncture path; IJV, internal jugular vein; CA, carotid artery.

The patient entered the operation room and inhaled pure oxygen via a mask. Routine monitoring of electrocardiogram (ECG), blood pressure (BP), heart rate (HR), and blood oxygen saturation (SpO_2_) was performed. All SGB operations were under the guidance of ultrasound, performed by the same senior doctor with expertise in ultrasound-guided technology (SonoSite M-Turbo, USA). The procedure for the modified C6 group was as follows: the patient was de-pillowed and tilted back, and the head was turned 45° to the left. The mouth was slightly opened to relax the anterior cervical muscles, and routine skin disinfection was performed. A high-frequency linear array probe (6–13 MHz) was utilized. The long axis of the probe was parallel to the plane of cricoid cartilage and formed an angle of 45° with the sagittal plane of the neck, which moved from the medial edge of sternocleidomastoid muscle to the outside. The ultrasound screen clearly displayed the C6 transverse process and its anterior and posterior tubercles. The anterior tubercle of the C6 transverse process is long; therefore, the ultrasonic image showed it as shallow. This often makes the gap between the anterior tubercle and the internal jugular vein too narrow (<5 mm), which affects the puncture (Figure 1). Therefore, for the modified C6 approach in this study, the ultrasonic probe was moved slightly toward C7, and when the anterior tubercle of the C6 transverse process disappeared from the ultrasound screen (Figure 2), the operator carefully observed the anatomical structures of the C6 nerve root, carotid artery, jugular vein, vertebral artery, thyroid, esophagus, and trachea, and assessed the distribution of blood vessels on the puncture path using an ultrasonic color technique. The needle was inserted through the gap between the C6 nerve root and the internal jugular vein using the in-plane technology. When the needle tip reached the surface of the long cervical muscle under the anterior fascia through the anterior scalene muscle, and when no blood, gas, and cerebrospinal fluid were observed during the withdrawal of the needle, 4 ml of 1% lidocaine was injected to complete the operation. For the C7 group, the procedure was as follows: the ultrasonic equipment, patient position, and preparations before block were the same as those in the modified C6 group. After the C6 transverse process was determined by the above method, the ultrasonic probe was moved parallel to the tail end to the level of the C7 transverse process (Figure 3). Then, after carefully identifying the anatomical structure, the needle was inserted through the gap between the C7 nerve root and the internal jugular vein to complete the block.

### Observation Indexes

(1) Block time: the duration from the ultrasonic probe first contacting the skin to the completion of SGB. (2) Number of puncture angle adjustments: the number of adjustments of needle angle required during SGB. (3) Block effects: the occurrence of Horner Syndrome after block indicated the success of the operation. Horner Syndrome is characterized by ipsilateral ptosis, pupil narrowing, blushing, elevated skin temperature, and nasal congestion, and the success rate of SGB was calculated on this basis. (4) Adverse reactions: occurrence of hoarseness, numbness of upper limbs, pneumothorax, hematoma, dizziness, slowed pulse, and other adverse reactions.

### Statistical Processing

Data were statistically analyzed using statistical software SPSS 23.0. The normally distributed measurement data for block time and number of puncture angle adjustments were expressed as mean ± standard deviation (X ± SD) and evaluated using *t*-testing. Count data for blocking effects and adverse reactions were expressed as percentage values (%) and compared using Chi-square testing. A *P* value of <0.05 was considered statistically significant.

## Results

Comparison of general conditions between the two groups. There was no significant difference in gender, age, weight and pain type between the two groups (*P* > 0.05), as shown in Table 1.

**Table 1:** Comparison of general conditions between the two groups (*n* = 65).

**Group**	***n***	**Age**	**Weight**	**Male: Female**	**Cervical spondylosis: headache: dysmenorrhea**
Modified C6 group	65	38.5 ± 10.2	62.8 ± 8.0	33:32	23:40:2
C7 group	65	38.2 ± 10.6	62.3 ± 8.2	35:30	25:37:3
t/χ^2^ value		0.164	0.352	0.031	0.475
*P* value		0.870	0.726	0.861	0.819

### Comparison of Block Time

The SGB operation time in the modified C6 group was shorter than that in the C7 group, and the difference between the two groups was statistically significant (*P* < 0.05: see Table 2).

**Table 2:** Comparison of block time between the two groups ($\bar{x}$ ± s,min).

**Group**	**Case**	**Block time**
Modified C6 group	65	5.1 ± 0.6
C7 group	65	8.2 ± 1.4
t value		16.409
p value		0.003

### Comparison of Number of Puncture Angle Adjustments

The number of puncture angle adjustments for the modified C6 group was smaller than that for the C7 group, and the difference between the two groups was statistically significant (*P* < 0.05: see Table 3).

**Table 3:** Comparison of the number of adjustments in the two groups of patients ($\bar{x}$ ± s,times).

**Group**	**Case**	**Number of adjustments**
Modified C6 group	65	1.8 ± 0.6
C7 group	65	3.2 ± 0.5
*t* value		18.581
*p* value		0.002

### Comparison of Block Effects

Horner Syndrome occurred after SGB in both groups, and the difference between the two groups was not statistically significant (*P* > 0.05: see Table 4).

**Table 4:** Comparison of blockade between the two groups [*n* (%)].

**Group**	**Case**	**Horner syndrome**
Modified C6 group	65	65 (100.00)
C7 group	65	65 (100.00)
χ^2^ value		0.000
*p* value		1.000

### Comparison of Incidence of Adverse Reactions

The incidence of adverse reactions in the modified C6 group was 4.62%, comprising 1 case of hoarseness and 2 cases of arrhythmia, while that in the C7 group was 6.15%, with 1 case of hoarseness and 3 cases of arrhythmia. The difference between the two groups was not statistically significant (*P* > 0.05: see Table 5).

**Table 5:** Comparison of adverse reactions between the two groups [*n* (%)].

**Group**	**Case**	**Hoarseness**	**Dizziness**	**Upper limb numbness**	**Pneumothorax**	**Hematoma**	**Slowed pulse**	**Total**
Modified C6 group	65	1 (1.54)	0 (0)	0 (0)	0 (0)	0 (0)	2 (3.08)	3 (4.62)
C7 group	65	1 (1.54)	0 (0)	0 (0)	0 (0)	0 (0)	3 (4.62)	4 (6.15)
χ^2^ value								1.163
*p* value								0.247

## Discussion

The stellate ganglion is a mixture of the 6th and 7th cervical ganglia fused with the 1st thoracic nerve node. It is located in the anterior area between the first rib neck and the base of the transverse process of the 7th cervical spine, covering the surface of the long cervical muscle on the deep surface of the anterior fascia. The important structures in the surrounding area include the vertebral artery, vertebral vein, phrenic nerve, and recurrent laryngeal nerve. In SGB, a local anesthetic is injected around the stellate ganglion on the deep surface of the anterior vertebral fascia, blocking the sympathetic activity dominated by the stellate ganglion (8). The mechanism of action of SGB is not yet fully understood: it may be related to its effective regulation of autonomic nerve and endocrine functions, thus inhibiting the function of the stellate ganglion, reducing sympathetic nerve activity, relieving cerebral vasospasm (9), improving local circulation and vascular compliance, and promoting an increase in cerebral blood flow (10).

SGB can be performed using traditional blind detection methods or guided by fluoroscopy or ultrasonic imaging (11). The stellate ganglion is deep, and the surrounding anatomical structures are complex. Adverse reactions such as hoarseness, numbness of upper limbs, pneumothorax, hematoma, dizziness, and arrhythmia can easily occur during block procedures performed using blind detection; therefore, accurate operation is particularly important. Ultrasound can display the soft tissue structures around the stellate ganglion clearly and allow for real-time dynamic observation of the running of the puncture needle and drug diffusion, thereby improving the effectiveness and safety of SGB (12). Ding et al. (2) reported that using ultrasound-guided positioning in SGB is more accurate and requires a lower dosage of local anesthetic than a blind detection approach, and that using ultrasound reduces injury, incidence of hematoma, pneumothorax, and other adverse reactions, and increases the safety of the procedure. Elmofty et al. (13) also reported that, compared with blind puncture, ultrasound-guided SGB greatly reduces the risk of stellate ganglion puncture and improves puncture success rate. Therefore, ultrasound-guided SGB has become the preferred method in clinical settings. Due to the different anatomical structures of the puncture planes, each potential SGB site has different important adjacent tissues; therefore, the difficulty of puncture operations varies greatly. The choice of puncture approach under ultrasound guidance is thus particularly important. There have been different reports regarding ultrasound-guided SGB through lateral approaches, including via the C7 transverse process and the C6 transverse process (3, 4). Because the anterior tubercle of the C6 transverse process is long (5), the gap between the anterior tubercle and the internal jugular vein is narrow (<5 mm), and this affects the needle entry. Therefore, this study innovatively adopted a modified C6 transverse process inferior approach to eliminate the influence of the C6 transverse process anterior tubercle on the puncture space. When the ultrasound screen clearly displayed the C6 transverse process and its anterior and posterior tubercles, the ultrasound probe was slightly moved toward C7, and the needle was inserted to complete the operation when the C6 transverse process anterior tubercle disappeared from the ultrasound screen.

There are different reports regarding the appropriate type and dosage of local anesthetic for SGB. Many countries most commonly use 0.25–0.375% bupivacaine, and 0.4–1% lidocaine is widely used in China. The injection volume of the anesthetic is generally 4–5 ml (14, 15). Yoo et al. (16) compared the SGB effects of 4-, 6-, and 8-ml dosses of local anesthetics and found that there was no significant difference. Another study (17) reported that ultrasound-guided SGB using 4 ml of 1% lidocaine had the same anesthetic effect and fewer side effects than with 6 and 8 ml.

In our study, ultrasound-guided puncture at the C6 transverse process level (modified) and the C7 transverse process level with 4 ml of 1% lidocaine successfully blocked the stellate ganglion, Horner Syndrome occurred, and the blocking success rate was 100%. These results are similar to those presented by Kim et al. (18). No upper limb numbness, pneumothorax, hematoma, or dizziness occurred, suggesting that ultrasound-guided SGB with 4 ml of 1% lidocaine is safe. This is related to the advantages of high-frequency ultrasound for clearly displaying the important soft tissue structures of the neck, guiding the puncture path in real time, and monitoring the diffusion range of the local anesthetic. There was one patient with hoarseness in each group; this was likely due to the local anesthetic spreading to the deep surface of thyroid to cause recurrent laryngeal nerve block in tracheoesophageal sulcus (4), indicating that the dose of lidocaine may need to be further reduced. Heart rhythm inhibition was the result of relative excitation of the vagus nerve after cervical sympathetic nerve block. This reaction was improved after symptomatic treatment with atropine, suggesting that contraindications such as sinus bradycardia and atrioventricular block should be strictly grasped.

In this study, the operation duration for C7 transverse process level SGB under ultrasound guidance was 8.2 ± 1.4 mins, and the number of puncture angle adjustments was 3.2 ± 0.5. For the modified C6 transverse process level SGB, the operation duration was shorter at 5.1 ± 0.6 mins, and the number of puncture angle adjustments was lower at 1.8 ± 0.6. These findings suggest that SGB at the modified C6 transverse process level is a quicker and less difficult operation; this is related to the different anatomical structures at the modified C6 and the C7 transverse process levels. Ultrasound images of the same patient at different puncture levels were collected to simulate the puncture paths (Figures 1–3). All the different approaches clearly showed the SGB area on the surface of the long cervical muscle under the prevertebral fascia. Because the anterior tubercle of the C6 transverse process is long, making the gap between the anterior tubercle and internal jugular vein narrow (<5 mm), the distribution of small blood vessels between spaces further affects the needle insertion angle (Figure 1). The modified C6 transverse process level puncture eliminated the influence of the anterior tubercle of the C6 transverse process, expanded the puncture clearance, reduced the number of needle insertion adjustments, and reduced the operation time (Figure 2). According to (19), the position at the C7 transverse process level is low and the surrounding tissue structures are complex; therefore, SGB carries a risk of penetrating the internal carotid artery and subarachnoid space, thus increasing the incidence of adverse reactions such as pneumothorax. A study in China (20) reported that, at the level of the C7 transverse process, the inferior thyroid artery tortuously runs on the surface of the long neck muscle, accounting for 31.2%, and the vertebral artery and vein and the ascending carotid artery also appear at the C7 transverse process level: similar findings are shown in the ultrasonic image in Figure 3. These factors affect the selection of the puncture path and increase the number of adjustments of the puncture needle, thus prolonging the operation time.

The limitations of this study were as follows: no self-control process of applying different approaches with the same patient was employed; Horner Syndrome was the only indicator used for assessing blocking success; the long-term therapeutic effects were not evaluated; and the number of cases was small.

In summary, the ultrasound-guided SGB method puncturing at the modified C6 transverse process level resulted in shorter operation time and a reduced number of puncture angle adjustments compared with the C7 transverse process approach. This modified method is thus worthy of clinical popularization and application.

## Data Availability Statement

The original contributions presented in the study are included in the article/supplementary material, further inquiries can be directed to the corresponding author/s.

## Ethics Statement

The studies involving human participants were reviewed and approved by this study was conducted in accordance with the declaration of Helsinki and approved by the Ethics Committee of the Second Affiliated Hospital of Soochow University (Ethics No.: JD-LK-2018-103-01). The patients/participants provided their written informed consent to participate in this study.

## Author Contributions

H-HS, H-FC, and X-LZ: conception and design of the research. H-FC and X-LZ: critical revision of the manuscript for intellectual content and writing of the manuscript. X-LZ: obtaining financing. H-FC: statistical analysis. J-XY: analysis and interpretation of the data. YN: acquisition of data. All authors read and approved the final draft.

## Conflict of Interest

The authors declare that the research was conducted in the absence of any commercial or financial relationships that could be construed as a potential conflict of interest.

## Publisher's Note

All claims expressed in this article are solely those of the authors and do not necessarily represent those of their affiliated organizations, or those of the publisher, the editors and the reviewers. Any product that may be evaluated in this article, or claim that may be made by its manufacturer, is not guaranteed or endorsed by the publisher.
